# Scleroderma Renal Crisis Debute with Thrombotic Microangiopathy: A Successful Case Treated with Eculizumab

**DOI:** 10.1155/2018/6051083

**Published:** 2018-10-23

**Authors:** Maite Hurtado Uriarte, Carolina Larrarte, Laura Bravo Rey

**Affiliations:** ^1^Nephrologist, University Hospital San Rafael, RTS Baxter, Bogota, Colombia; ^2^Nephrologist, University Hospital Militar, RTS Baxter, Bogota, Colombia; ^3^Medical student, Javeriana University, Bogota, Colombia

## Abstract

We had the challenged to treat a 40-year-old female with Systemic Scleroderma who was showing unspecific symptoms. During her time at the hospital she rapidly develops renal dysfunction, associated with hypertension. She required renal replacement therapy initiation and we observed a decline in hemoglobin and platelets numbers. We confirm a microangiopathic hemolytic anemia and rule out other immune diseases or thrombotic thrombocytopenic purpura. Systemic Sclerosis is a chronic immune disorder of unknown origin that it is not completely understood. It is believed that environmental factors may contribute and also altered genes may be implicated in the immune system's function. Microangiopathic hemolytic anemia occurs in 43% of patients who develop scleroderma renal crisis and an activation of the complement system through the classical pathway may be involved. Given that context we decided to treat the patients with C5 blocker Eculizumab and obtain an extraordinary positive response.

## 1. Introduction

Systemic sclerosis (SSc) is a chronic and complex autoimmune disorder that it is not fully understood. It is characterized by microvascular endothelial cell apoptosis, excessive extracellular matrix protein deposition and perivascular infiltration of mononuclear cells, producing damage and progressive fibrosis of the skin and visceral organs (lungs, heart, and kidney). It has been divided into two categories; (i) limited and (ii) diffuse [1–3]. The first affects only the skin of distal extremities and face, usually characterized by a very slow clinical course. On the other hand, the diffuse type affects wide areas of skin and internal organs. Neither category has completely effective treatment available, mainly as a result of the lack of knowledge of its pathogenesis, which is an obstacle for the prescription of the right treatment or at least for taking the appropriate measures to slow down its adverse effects [4].

It has been seen that environmental factors may contribute [5]; immune genes are also involved in the abnormal regulation of T and B cells and the activation of the complement system through the classical pathway, which targets the vascular and connective tissue [6–8].

Scleroderma renal crisis is defined by: first, severe or worsening arterial hypertension, and second a quick and progressive kidney failure, in both cases in the absence of any other case [9].

The most recent data shows that renal crisis is found in 1% of cases with limited scleroderma; on the other hand, in the diffuse type it was found between 4 to 11% [10] and in some reports up to 25% [9].

**Figure 1:**
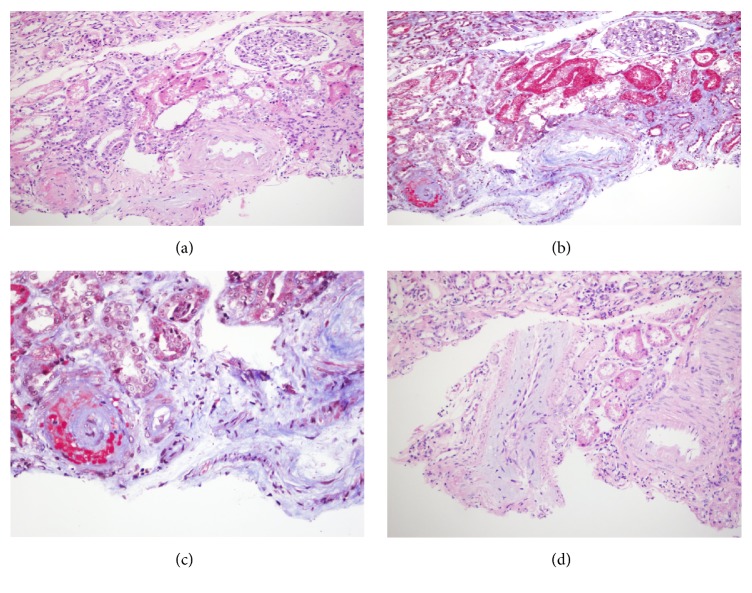
Renal parenchyma with glomerular ischemia. ((a) Hematoxylin y Eosin 10X, (b) Trichrome 10X), associated with vascular changes in interlobular arteries: fibrinoid necrosis of the intima with fragmentation of erythrocytes, ((c) Tricromico 20X) and mucoid expansion of the intima with luminal obliteration, and ((d) haematoxylin and eosin 10X).

**Figure 2:**
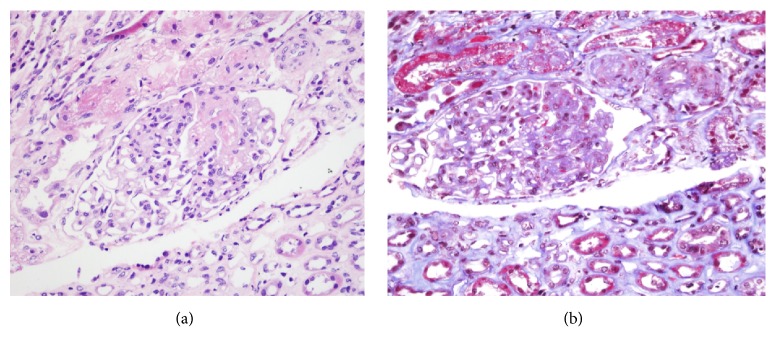
Glomerulus with subendothelial edema towards the vascular pole and arterioles with luminal obliteration due to endothelial edema and fibrinoid necrosis. ((a) Hematoxylin and 40X eosin and (b) Trichrome 40X).

Abnormalities found on real biopsy are “onion bulbs” in the arteries (Figures 1-2) caused by endothelial injury, in which intimal proliferation and vascular remodeling lead to the obstruction of the vascular lumen and the reduction of glomerular filtration rate [10].

## 2. Case Presentation

**Figure 3:**
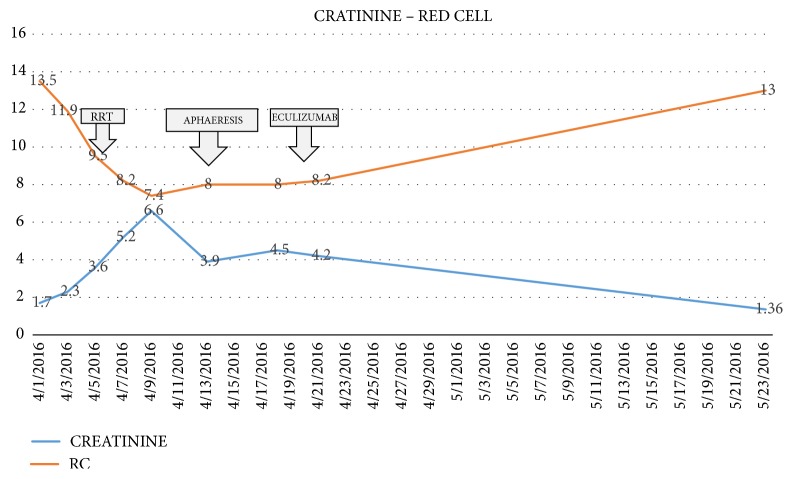
Initial increase of creatinine levels showing decrease of renal function and decrease of red blood cells, hemolytic anemia, the breaking point on day 9, after 10 days after trying with RRT and aphaeresis Eculizumab therapy was started with a subsequently recovery of red cell line and creatinine level.

**Figure 4:**
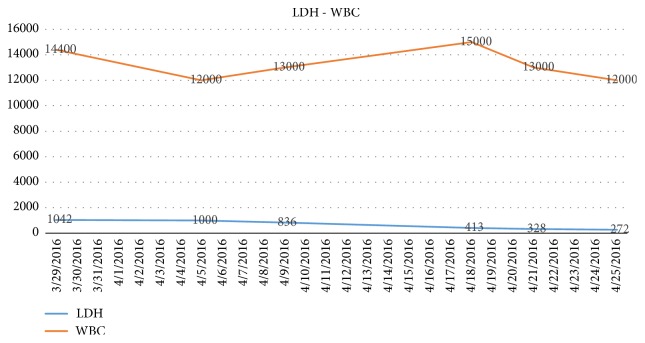
We keep track of white blood cells and LDH showing the impact on all blood lines and hemolytic anemia.

**Figure 5:**
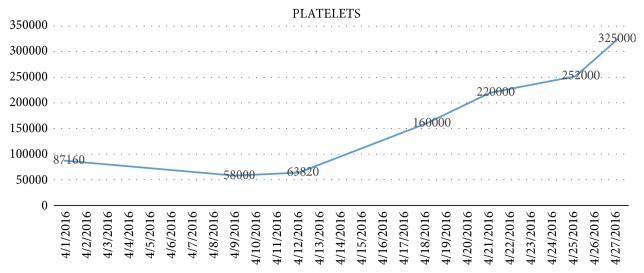
This graphic shows the platelet count decrease at the beginning of the disease; that is the reason of suspect Thrombotic thrombocytopenic purpurea; we can see the ascending curve after day 19 with the start of Eculizumab therapy.

We present the case of a 40-year-old woman with a history of systemic sclerosis, diagnosed 3 years ago. She arrived without treatment, due to a poor toleration of the medication metrotexate. She requested medical help in different opportunities for unspecific symptoms for 3 months including; nausea, vomiting, dizziness, asthenia and loss of weight. She didn't demonstrate any improvement and arrived with an uncertain diagnosis. Our institution observed symptoms, showing a decline in her renal function (creatinine 1,6 mg/dl and Uremic nitrogen blood BUN 41,3), with a urine test showing hematuria 28 xc, associated with hypertension 214/140 mmHg. Initially she was treated for an infection, showing rapid renal deterioration to creatinine 6,67 mg/dl and BUN 96,77 mg/dl with oliguria and overload. We started conventional treatment with IECA and calcium channel blockers, the patient showed no response, on the contrary her renal functiondeclined to the point that RRT was needed (Figure 3). At the same time, the patient developed a deep thrombocytopenia and anemia, showing smear schistocytes in the blood, elevated Lactate Dehydrogenase (LDH), and consumption of haptoglobin (Figures 4-5.) We ruled out Thrombotic Thrombocytopenic Purpura (TTP) because ADAMST 13 was normal; we also dismissed different immunologic disease. Kidney histopathology showed Thrombotic Microangiopathy (TMA), therefore we started plasma exchange getting slight improvement. Our patient showed a dramatic decline in renal, hematologic, and cardiac functions, therefore we decided to initiate treatment using C5 blocker with previous vaccination against encapsulated bacteria, resulting in an improvement in platelet count and red cells within the first week. 6 months later the patient showed full renal recovery and as a result RRT treatment was no longer needed.

Given the dramatic morbidity and mortality of this disease, in particular in the context of our incomplete understanding of its roots, we believe that to present this case may be relevant as it shows a successful outcome that may lead to new ways to approach the research needed to develop more suitable methods and treatments.

## 3. Discussion–Conclusions

Our understanding of the pathogenesis of renal damage in systemic sclerosis remains incomplete; however it seems to result from a series of factors affecting the kidney. The primary process is the injury of the endothelial cells, which results in intimal proliferation of renal arteries (Figures 1-2). We do not know with certainty the physiopathology, but recent findings suggest that an activation of the complement system through the classical pathway may be are involved. It is believed that certain proteins are involved in either promoting or maintaining an inflammatory state, such as a variation on factor H as associated with endothelial cells damage [11, 12].

The renin Angiotensin System plays an important role in this complication. That is why ACE inhibitors alter survival substantially. Before the use of this, less than 10% of patients survived the first year; after the use of this drug, 5-year survival increased to 65%. Nevertheless, almost 40% of the patients are still going to require dialysis, and 25% will die within one year [9].

As microangiopathic hemolytic anemia occurs in 43% of patients who have scleroderma renal crisis, it has been suggested that an activation of the complement system through the classical pathway may be involved.

As a result of the dramatic clinical and histological severity and the lack of responses to the conventional treatment; IECA, calcium channel blockers, and plasma exchange, recently the use C5 blocker eculizumab has been proposed.

Eculizumab is a humanized recombinant immunoglobulin G2/4 monoclonal antibody directed against the complement component C5; by binding to complement component C5, the drug inhibits the generation of C5a and C5b-9, and thus subsequently inhibits the lysis and endothelial damage.
